# Integrative stress management for global workforce: music-based and exercise intervention for overseas employees

**DOI:** 10.3389/fpubh.2025.1603059

**Published:** 2025-11-06

**Authors:** Yaming Wei, Huijuan Wu, Yongfan Song

**Affiliations:** 1 Research Center for Arts and Health, Xiamen Humanity Hospital, Xiamen, Fujian, China; 2 School of Humanities and Social Sciences, Fuzhou University, Fuzhou, Fujian, China; 3 Department of Physical Education Research, Fuzhou University, Fuzhou, Fujian, China

**Keywords:** expatriate workers, music-based interventions, exercise intervention, occupational stress, mental health

## Abstract

**Background:**

Overseas employees experience high stress from cultural, work, and social challenges. This study examines whether combining music-based intervention and aerobic exercise reduces stress, and explores the influence of age, occupation, music preference, and long-term effects.

**Methods:**

We conducted a randomized trial with 92 Chinese expatriate workers, randomly assigned to either (1) an experimental group receiving weekly 45-min resource-oriented music-based intervention sessions plus 30-min moderate-intensity exercise, or (2) a control group receiving placebo interventions (background music + stretching) for 5 weeks. Stress levels were assessed using the Global Assessment of Recent Stress (GARS) at baseline (pre-intervention), post-intervention (0 months), and at 1-, 3-, and 6-month follow-ups through online surveys, supplemented by qualitative interviews.

**Results:**

The experimental group demonstrated significant stress reduction (GARS scores decreased from 3.59 ± 0.71 to 2.68 ± 0.57, *p* < 0.001), with effects sustained at 6 months (*p* < 0.05). Subgroup analyses showed enhanced efficacy for (a) employees aged 40–49 years, (b) classical music listeners, and (c) high-stress occupations like seafarers. Qualitative data corroborated these findings by highlighting participants’ subjective experiences of improved emotional regulation, reduced anxiety, and increased relaxation, aligning closely with the quantitative stress reduction outcomes.

**Conclusion:**

This dual-modality intervention effectively reduces stress among overseas workers, with benefits influenced by individual characteristics. The results support implementing personalized, non-pharmacological stress management programs in multinational corporations to promote global workforce mental health.

## Introduction

In recent years, with the acceleration of globalization, an increasing number of Chinese employees have chosen to work overseas (1, 2), making significant contributions to corporate internationalization and the implementation of China’s “Belt and Road” initiative (3). However, the complexity of overseas work environments has also imposed unprecedented psychological stress on employees (4, 5). Research indicates that challenges such as cultural adaptation, language barriers, separation from family and friends, and the high intensity and risk associated with job responsibilities contribute to significantly higher stress levels among overseas employees than their domestic counterparts (6, 7). Moreover, social isolation and a lack of local support systems further exacerbate mental health issues (8). Prolonged exposure to high stress harms employees’ physical and mental health, significantly reduces work efficiency, increases absenteeism, and elevates turnover rates. Existing studies have shown that psychological stress can lead to anxiety, depression, and other mental health issues, as well as physiological conditions like cardiovascular diseases, even posing life-threatening risks (9). Therefore, providing effective stress management strategies for overseas employees has become an urgent priority for enterprises and academic researchers.

Occupational stress is strongly associated with increased absenteeism, decreased job satisfaction, lower performance, and heightened turnover intentions, thereby undermining organizational productivity and efficiency (10). Economic stress further amplifies these effects by reducing employees’ innovation and work engagement through absenteeism (11). Another subtle yet impactful issue is presenteeism—employees being physically present but mentally or emotionally disengaged—resulting in reduced team performance and productivity (12). Furthermore, job stress correlates negatively with job satisfaction, which in turn reduces employee loyalty, motivation, and organizational commitment (13). These risks are particularly pronounced in high-stress occupations such as healthcare, logistics, and maritime shipping, where employees are more susceptible to burnout, absenteeism, and depression, especially during crises like the COVID-19 pandemic (12). Although awareness of workplace mental health has grown, current interventions are often short-term and lack individualization. Therefore, there is an urgent need to develop effective, sustainable, and culturally adaptable strategies for stress management among expatriate employees.

Music-based interventions have emerged as diverse non-pharmacological approaches in the field of mental health (14). By delivering structured musical stimuli, they help regulate emotions through mechanisms such as activation of the limbic system and reduction of cortisol levels (15). This approach is based on the emotional regulation functions of music, which improve emotional states by influencing the central nervous system (14, 16–18). Studies have demonstrated that resource-oriented music therapy effectively reduces stress-related hormones, such as cortisol, promotes relaxation, and improves sleep quality (19, 20).

Systematic reviews have shown that music-based interventions can reduce salivary cortisol levels, possibly through enhanced parasympathetic activity (19). This study is grounded in Resource-Oriented Music Therapy (ROMT), which promotes mental health by activating internal resources such as cultural identity, aesthetic preferences, and emotional memory, rather than focusing solely on deficits. Unlike traditional pathology-based models, ROMT adopts a strengths-based approach using techniques like improvisation and analysis of preferred music to support self-healing (21). Personalized music has shown greater effects in reducing state anxiety than standardized playlists, especially in populations facing cross-cultural adaptation stress (22). However, with limited evidence for transnational employees, most existing research has focused on perioperative patients and older adults (23, 24). Thus, evaluating the effectiveness of music-based interventions in stress management for overseas workers is both necessary and timely.

Aerobic exercise is an important physiological intervention for stress management, regulating cortisol secretion via activation of the hypothalamic–pituitary–adrenal (HPA) axis (25). Recent research supports the utility of diurnal salivary cortisol patterns as indicators of regulatory function within the HPA axis, particularly in relation to stress adaptation and adrenal sensitivity (26). When combined with music listening, rhythmic auditory stimuli can enhance exercise adherence, while exercise-induced endorphin release and music-induced dopaminergic activation may produce synergistic effects (27, 28). This dual-modality approach engages emotional regulation through the limbic system and autonomic balance through physical activity (29, 30). In addition to cortisol, heart rate variability (HRV) is increasingly recognized as a reliable non-invasive biomarker of autonomic nervous system activity and stress reactivity. Decreased HRV reflects reduced parasympathetic tone and heightened stress responses, making it a valuable physiological indicator in emotion regulation and music intervention studies (31). However, such combined interventions are underexplored, particularly among overseas employees. Most existing studies emphasize short-term outcomes, with limited evidence of sustained effects. Further research could provide new perspectives for psychological health interventions.

This randomized trial evaluates the effectiveness of a combined music-based intervention and moderate-intensity aerobic exercise in reducing stress among overseas employees. In our current study, a triadic moderation model incorporating age, occupation, and music preference is proposed to examine the role of personalized factors. Long-term follow-up addresses the current gap in evidence regarding the durability of such composite interventions. Building on existing literature in psychophysiology and music therapy, this study applies a dual physiological-psychological perspective to interpret the synergistic mechanisms of the intervention, focusing on neuroendocrine regulation and behavioral pathways. These findings provide empirical support for developing cross-culturally adaptive stress management strategies in the workplace and offer new directions for non-pharmacological mental health interventions.

## Materials and methods

### Participants

**Figure 1:**
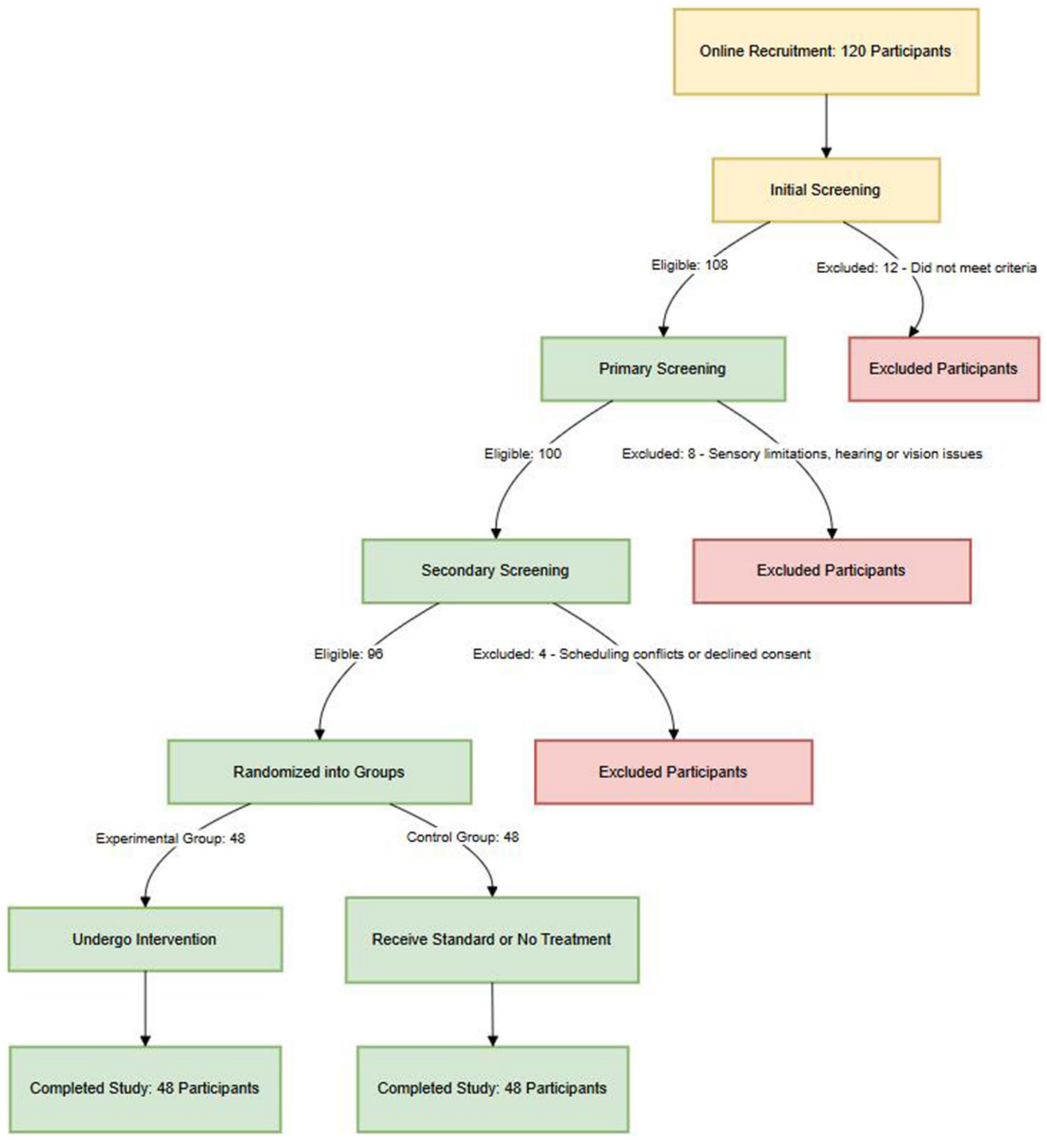
Flowchart of participant inclusion and exclusion in the resource-oriented music therapy and exercise interventions study.

This randomized controlled trial (RCT) was conducted online, with participant recruitment carried out in collaboration with the human resources departments of large Chinese multinational corporations. Recruitment was conducted online through internal corporate platforms, WeChat groups for expatriate employees, and LinkedIn. Inclusion criteria were as follows: (1) Chinese nationality with over 1 year of overseas work experience; (2) self-reported stress-related issues; and (3) no significant hearing or visual impairments. Based on a previous study on music therapy for stress reduction (32), a medium effect size (Cohen’s *d* = 0.5) was assumed. With a two-tailed independent samples t-test, *α* = 0.05, and statistical power set at 0.80, the estimated minimum required sample size was 45 participants per group. Considering an anticipated attrition rate of approximately 10%, the target sample size was set at 92 (46 per group). A total of 128 overseas employees from Chinese enterprises enrolled in the combined music-based and moderate-intensity aerobic exercise intervention program (Figure 1). After screening, 92 eligible participants were included and randomly assigned to the experimental and control groups (*n* = 46 each) using a computer-generated randomization sequence, meeting the statistical power requirement. Randomization was performed using a computer-generated random number table to ensure allocation concealment and minimize selection bias. Due to COVID-19 restrictions and time zone differences, all procedures were conducted remotely via Zoom, including informed consent, baseline assessments, and intervention delivery.

### Research methods and procedures

Participants were randomly assigned to the experimental or control group. The control group received a placebo intervention consisting of weekly exposure to non-personalized background music, such as standardized piano pieces or natural ambient sounds with consistent tempo and minimal emotional variation (Supplementary Table S1), followed by 30 min of low-intensity physical activity. Exercises included neck rolls, shoulder rotations, lateral trunk bends, leg stretches, and simplified standing balance movements. These activities emphasized flexibility and gentle movement, with heart rate maintained below 50 percent of the estimated maximum. All sessions were supervised by trained assistants.

The experimental group received a five-week intervention comprising a weekly 45-min personalized music therapy session (Supplementary Table S2) followed by 30 min of moderate-intensity aerobic exercise. Exercise activities included brisk walking, step routines, balance training, and foundational yoga poses such as Tadasana, Cat-Cow, and Bridge. To control for order effects, the intervention followed a fixed sequence in which music therapy was conducted first, followed by aerobic exercise. This structure was based on the relaxation-before-activation principle, which aims to reduce autonomic arousal through music and establish a physiological and psychological foundation for subsequent physical activity (33). A five-minute rest period was included between components to facilitate the transition. The control group followed the same structure, with background music preceding stretching and movement, to ensure consistency in intervention timing and format and to minimize potential procedural confounds.

Music selections were based on individual preferences collected during baseline assessment (34), drawn from a standardized music library, and adjusted in tempo according to real-time HRV. During the intervention, HRV was continuously monitored using wearable devices to assess participants’ autonomic responses. The HRV data informed real-time adjustments to the music intervention, aiming to enhance relaxation and stress reduction. The aerobic exercise component included brisk walking or yoga, with metronome-guided audio used to help maintain the target heart rate zone. The control group received placebo interventions involving non-personalized background music and light stretching exercises. All interventions were delivered once per week over 5 weeks.

**Figure 2:**
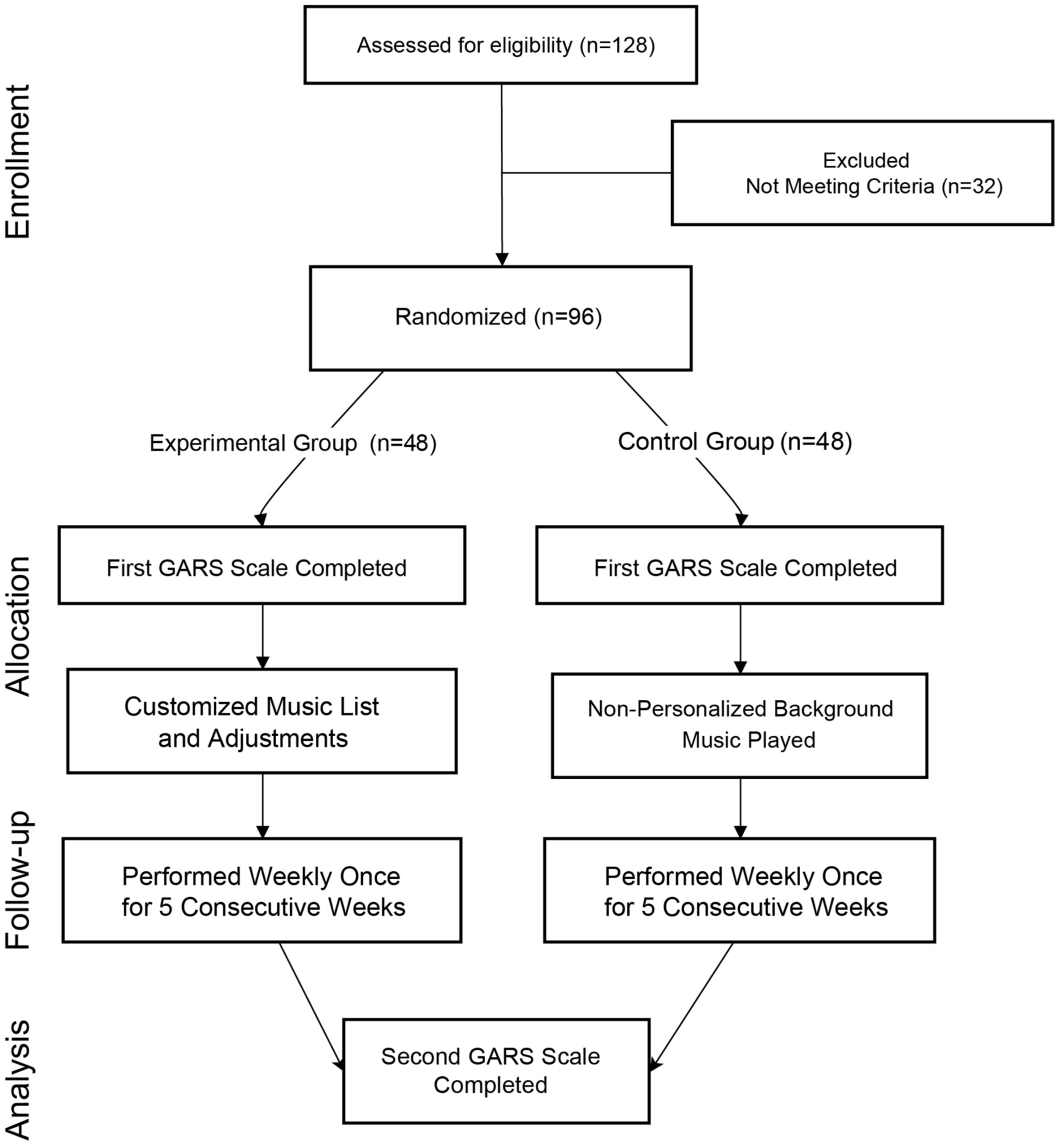
Flow diagram of the treatment process.

As illustrated in Figure 2, at the beginning of the study, participants completed a demographic questionnaire, including music preferences. Certified music therapists (MT-BC), accredited by the American Music Therapy Association, developed individualized treatment plans and made adjustments during the sessions as needed. Each music session involved live music and singing, and participants were instructed to avoid other activities during the session to maintain full engagement.

The intervention was evaluated across three dimensions. Perceived stress was measured using the Global Assessment of Recent Stress (GARS) (35) at five time points: baseline (pre-intervention), post-intervention (0 months), and at 1-, 3-, and 6-month follow-ups through online surveys. Salivary cortisol levels were collected using mail-in sampling kits and analyzed to evaluate HPA axis activity as a physiological marker of stress. Exercise adherence was monitored using Fitbit wristbands provided uniformly by the research team. Adherence was defined as achieving an average of at least 8,000 steps per day. To ensure balanced allocation between the experimental and control groups, block randomization was conducted using the REDCap (Research Electronic Data Capture) system (36). A block size of four was applied, meaning every four consecutively enrolled participants were randomly assigned, in a preset sequence, to two participants per group. This approach maintained group balance throughout recruitment and is particularly suitable for randomized controlled trials with moderate sample sizes, minimizing allocation bias. Randomization was performed by the research team in a blinded manner to preserve allocation independence and objectivity. Outcome assessors were blinded to group assignments, and the visual interfaces for both interventions were kept consistent to control for expectation-related bias.

### GARS

The GARS is a valuable tool for measuring individual stress levels across various environments, including music-based intervention settings (35). By conducting GARS assessments before and after music-based intervention sessions, therapists can better understand the effects of resource-oriented music therapy listening on reducing clients’ stress levels. This study used changes in GARS scores to identify factors influencing participants’ overall stress levels and specific stressors. The GARS items were adapted to align with the study’s objectives (Supplementary Figure S1). Participants were asked to rate the frequency and intensity of each stressor on a 5-point scale, ranging from “never” to “always” for frequency and “not stressful at all” to “extremely stressful” for intensity. Based on the scale, personal information has been added.

### Resource-oriented music therapy listening program

Resource-oriented music therapy listening is a music-based intervention approach designed to discover and utilize the internal resources of patients to promote recovery and growth. This study’s resource-oriented music-listening method includes five steps (Supplementary Table S3). The first step is the preparation phase, which involves collecting personal information and signing the agreement to participate in the experiment. This step aims to understand the participants and lay the foundation for the music-listening intervention. The second step was a preliminary assessment conducted by a board-certified RMT to evaluate participants’ stress levels. The music therapist calculates the total score based on the participants’ completion of the five-point GARS assessment. This step helps the therapist understand the participants’ stress levels and guide the development of the music-based intervention plan. The third step is the music intervention, which alleviates overseas employees’ work and life stress through music-based interventions. The intervention consists of a private music-based intervention program conducted over five consecutive weeks, with one 45-min session per week. The program may include discovering internal resources, sharing music, selecting music matching the internal resources, resource-oriented guidance, and listening to music. In the discovering internal resources phase, the music therapist assists participants in identifying their internal resources, including self-awareness, coping skills, and resilience. Participants are encouraged to share their music preferences in the sharing music phase, providing insights into their emotions and experiences. The internal resource matching phase involves selecting music that corresponds to the internal resources identified in the first phase. Finally, the resource-oriented guidance phase helps participants learn how to use their internal resources to manage stress and promote health. The fourth step is the reassessment, where participants’ stress levels are re-evaluated after the five music-based intervention sessions. The total score of the five-point GARS assessment is calculated to evaluate the effect of the music-based interventions intervention. This step objectively measures stress level improvement and informs further treatment plans. The fifth step is evaluating the effectiveness of the music-based interventions, conducted through one-on-one interviews. The purpose is to investigate participants’ entire treatment process and resource orientation. During the interviews, participants are asked about their experiences, emotions, and the effects of the music-based interventions intervention.

### Follow-up

The follow-up assessments were conducted at 1 month, 3 months, and 6 months after the intervention. Data were collected through online surveys, utilizing the GARS to evaluate stress levels at each time point. No instances of data loss or participant dropout were reported during the follow-up period, ensuring the completeness and reliability of the collected data.

### Data collection and analysis

Data were collected at two-time points: before and after the five-week intervention. Participants completed the standardized five-point GARS questionnaire to quantify stress levels, supplemented by one-on-one in-depth interviews to obtain qualitative data. All participants provided written informed consent before participation, with clear communication of voluntary involvement, data confidentiality, and the right to withdraw at any time. The GARS was administered at five time points: baseline (pre-intervention), post-intervention (0 months), and at 1-, 3-, and 6-month follow-ups to assess changes in perceived stress over time. Post-intervention interviews explored participants’ subjective experiences, emotional changes, and perceptions of the intervention’s impact, providing contextual support for the quantitative findings.

All data were double-entered and cross-verified for consistency. Outliers were addressed before analysis. Statistical analyses were conducted using SPSS version 27. Data normality was tested using the Shapiro–Wilk and Kolmogorov–Smirnov tests (*p* > 0.1). Non-normal variables were log-transformed. Gender and history of mental illness, as categorical variables, were compared between groups using chi-square tests. Continuous variables including age, years working overseas, coded profession scores, and baseline perceived stress were analyzed using independent samples t-tests. Within-group differences in stress were analyzed using paired t-tests (df = 45, 95% CI) and between-group differences using independent samples t-tests with Hedges’ g correction (df = 90). Time effects were evaluated using repeated measures ANOVA with Greenhouse–Geisser correction. Moderating effects of age, occupation, and music preference were examined using hierarchical regression analysis (Δ*R*^2^ > 5% indicating clinical relevance). Sensitivity analyses included Bland–Altman plots, and Mann–Whitney U tests. Effect sizes were interpreted according to Cohen’s criteria (Cohen’s *d* ≥ 0.8, indicating a large effect). All statistical codes and datasets are available upon request.

## Results

### Baseline characteristics comparison

Table 1 presents the experimental and control groups’ baseline demographic characteristics and pre-intervention stress levels. In the experimental group, there were 33 males and 13 females, while the control group included 37 males and 9 females. The difference in gender distribution between groups was not statistically significant (*χ*^2^ = 0.73, *p* = 0.393). The mean age was identical across groups at 32.89 years (SD = 5.413) (*t* = 0.000, *p =* 1.000). The mean occupational code was 2.63 in the experimental group and 2.89 in the control group. Average years of overseas work experience were 3.22 for the experimental group and 3.83 for the control group. These differences were not statistically significant (*p* > 0.05). Regarding history of mental illness, 4 participants in the experimental group and 1 participant in the control group reported having a history. The between-group difference was not statistically significant (*χ*^2^ = 2.030, *p* = 0.154). Pre-intervention stress levels, as measured by the Global Assessment of Recent Stress (GARS), showed a mean score of 3.59 (SD = 0.71) in the experimental group and 3.65 (SD = 0.63) in the control group (*t* = 0.435, *p =* 0.664). The experimental group comprised 72% male participants, and the control group had 80% male participants. Regarding occupational composition, 42.4% of participants were in technical roles and 35.9% in project management positions.

**Table 1:** Demographic characteristics and pre-test perceived pressure assessment of participants.

Demographic characteristics	Experiment group	Control group	Difference	
	*M* (SD)	*M* (SD)	Test	*p*-value
Gender	Male = 33 Female = 13	Male = 37 Female = 9	*χ*^2^ = 0.730	0.393
Age	32.89 (5.413)	32.89 (5.413)	0.000	1.000
Number of years working overseas	3.22 (2.149)	3.83 (2.058)	1.387	0.169
History of mental illness	Yes = 4 No = 42	Yes = 1 No = 45	*χ*^2^ = 2.030	0.154
Profession	2.63 (1.181)	2.89 (0.900)	1.192	0.237
Pre-test				
Perceived pressure assessment	3.59 (0.71)	3.65 (0.63)	0.435 (90)	0.664

Gender and history of mental illness were analyzed using chi-square tests due to their categorical nature, while continuous variables such as age, years working overseas, profession (coded), and pre-test stress scores were compared using independent samples t-tests.

### Analysis of the occupational background and overseas work experience of the experimental group participants

This section reports the occupational backgrounds and overseas work experience of participants in the experimental group (Table 2). Among the 46 participants, 33 were male and 13 were female. The age range was 23–48, with a mean age of 32.9 and a median age of 32. Overseas work experience ranged from 1 to 10 years, with a mean of 3.2 and a median of 3 years.

**Table 2:** Demographic information of participants.

Variable	Category	Count (*n*)
Gender distribution	Male	33
	Female	13
Age distribution	20–29	14
	30–39	25
	40–49	7
Range	23–48	
Mean age	32.9	
Median age	32	
Overseas work years	1	10
	2	12
	3	7
	4	6
	5	7
	8	3
	10	1
Range	1–10	
Mean years	3.2	
Median years	3	
Daily working hours	–	10
Psychiatric history	Male	2
	Female	2
Profession distribution	Manager	2
	Seafarer	13
	Legal Specialist	3
	Salesman	9
	Technician	7
	Bank Staff	2
	Logistical Personnel	10
Total	Total	46

Occupational categories included 2 managers, 13 seafarers, 3 legal professionals, 9 sales staff, 7 technicians, 2 bank employees, and 10 logistics and customer service personnel. Four participants (2 male and 2 female) reported a history of mental health conditions, including anxiety and depression. The average reported working hours per day was 10 h.

### The significant effect of resource-oriented music therapy in reducing seafarers’ stress levels

This study collected and analyzed Global Assessment of Recent Stress (GARS) scores before and after the intervention. The experimental group had a mean pre-intervention score of 3.59 (SD = 0.71), which decreased to 2.68 (SD = 0.57) after the intervention. The control group’s pre-intervention mean score was 3.65 (SD = 0.63), and the post-intervention score was 3.52 (SD = 0.64). Independent-sample t-tests showed a statistically significant difference between the post-intervention scores of the two groups (*t* = −6.633, *p* < 0.001), with a partial *η*^2^ of 0.328, indicating a moderate effect size (Table 3).

**Table 3:** Comparison of GARS pre-test and post-test scores between groups.

Measure	Group	Mean	Std. Deviation	Std. Error Mean	*t*	*p*-value
Pre-test score	Experimental group	3.593	0.706	0.104		
	Control group	3.654	0.632	0.093	0.435	0.664
Post-test score	Experimental group	2.685	0.57	0.084		
	Control group	3.522	0.639	0.094	6.381	<0.001

**Figure 3:**
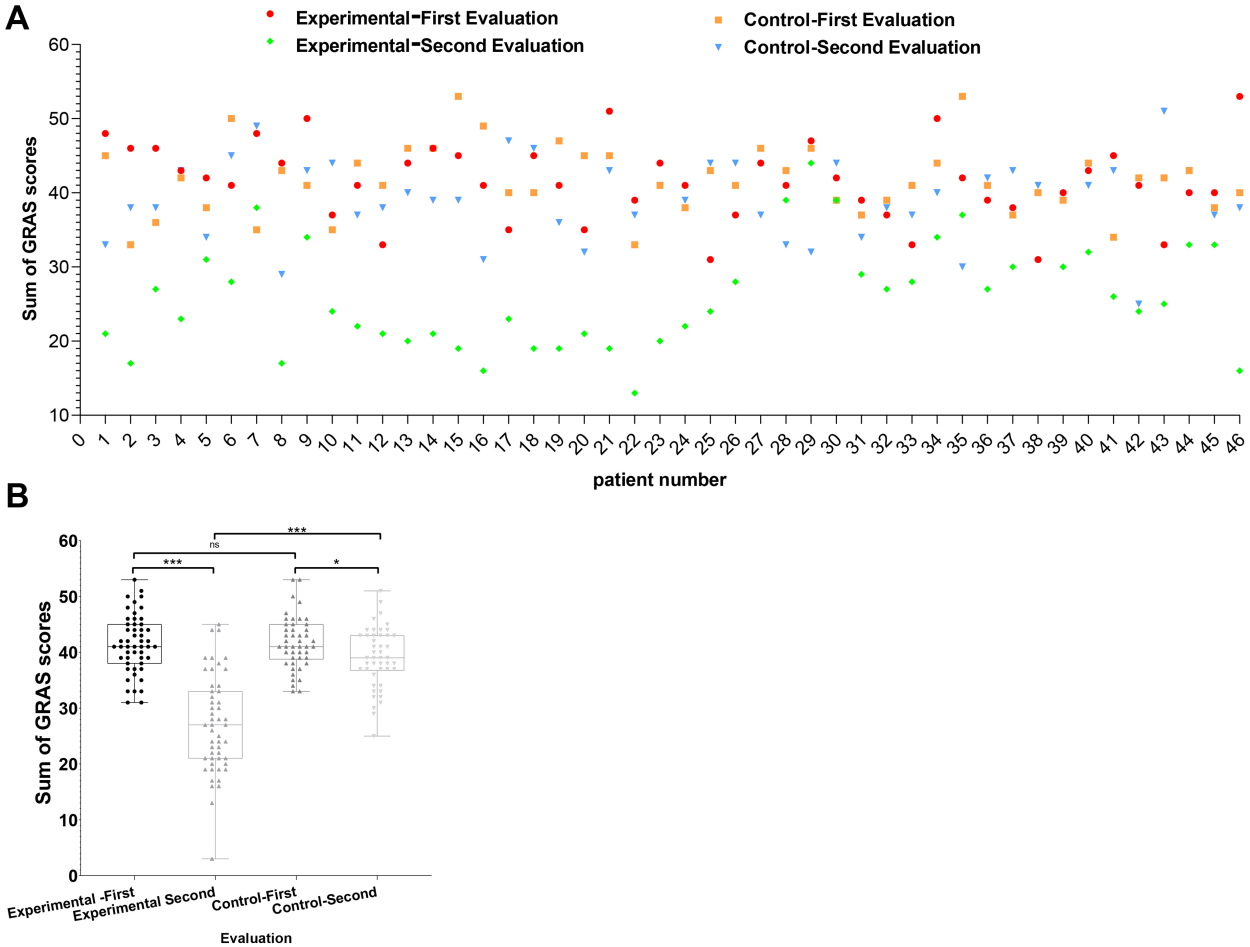
Visualization of changes in work-related stress among participants before and after resource-oriented music-based interventions. **(A)** Scatterplot of GARS scores for experimental (*n* = 46) and control groups (*n* = 46) recorded during the first and second evaluations. Red and green dots represent the first and second evaluations for the experimental group, respectively, while orange squares and blue triangles represent the first and second evaluations for the control group. **(B)** Box plot comparing GARS scores between groups before and after the intervention. Four categories are depicted: first and second evaluations for both the experimental and control groups. Statistical comparisons were conducted using independent sample t-tests, with significance levels indicated as *p < 0.05, **p < 0.01, and ***p < 0.001.

Individual changes in stress levels are shown in Figure 3A. The experimental group displayed overall decreases in GARS scores, with some participants showing larger reductions. The control group showed relatively stable scores across the intervention period. Figure 3B presents a boxplot comparison of pre- and post-intervention scores. The experimental group’s median score decreased after the intervention, with a concurrent reduction in score variability.

To complement the subjective GARS assessments, salivary cortisol samples were collected from all 92 participants at both baseline and immediately after the five-week intervention. However, due to temperature fluctuations during international mailing, a portion of the samples degraded and did not pass laboratory quality control. As a result, valid paired cortisol data were not sufficient for inclusion in statistical analysis. Preliminary inspection of the available samples suggested a downward trend in the experimental group, while the control group remained relatively stable. Although not analyzed quantitatively, this observed pattern aligns with theoretical models of HPA axis modulation and supports further physiological investigation in future studies.

### Greater stress relief observed in participants aged 40–49

A subgroup analysis was conducted based on age groups within the experimental cohort to further examine the influence of age on the outcomes of resource-oriented music therapy interventions (Table 4).

**Table 4:** Age on therapeutic music listening in stress relief of overseas dispatched employees.

Age group	Measurement stage	Number	Min	Max	25% Percentile	75% Percentile	Mean ± SD	S.E. Mean	LCL	UCL	Med
20–29	Pre-test	13	31	46	38.5	44	40.46 ± 4.18	1.158	38	45	41
	Post-test		19	33	21.5	31	26.77 ± 5.00	1.387	21	31	27
30–39	Pre-test	26	31	53	37	45.25	41.19 ± 5.70	1.118	37	44	41
	Post-test		16	44	21	34	27.35 ± 8.29	1.626	22	33	24.5
40–49	Pre-test	6	39	51	40.5	48.75	45.17 ± 4.62	1.887	39	51	44
	Post-test		13	38	16	30.5	22.67 ± 9.00	3.676	13	38	20

LCL, Lower Confidence Limit; UCL, Upper Confidence Limit.

**Figure 4:**
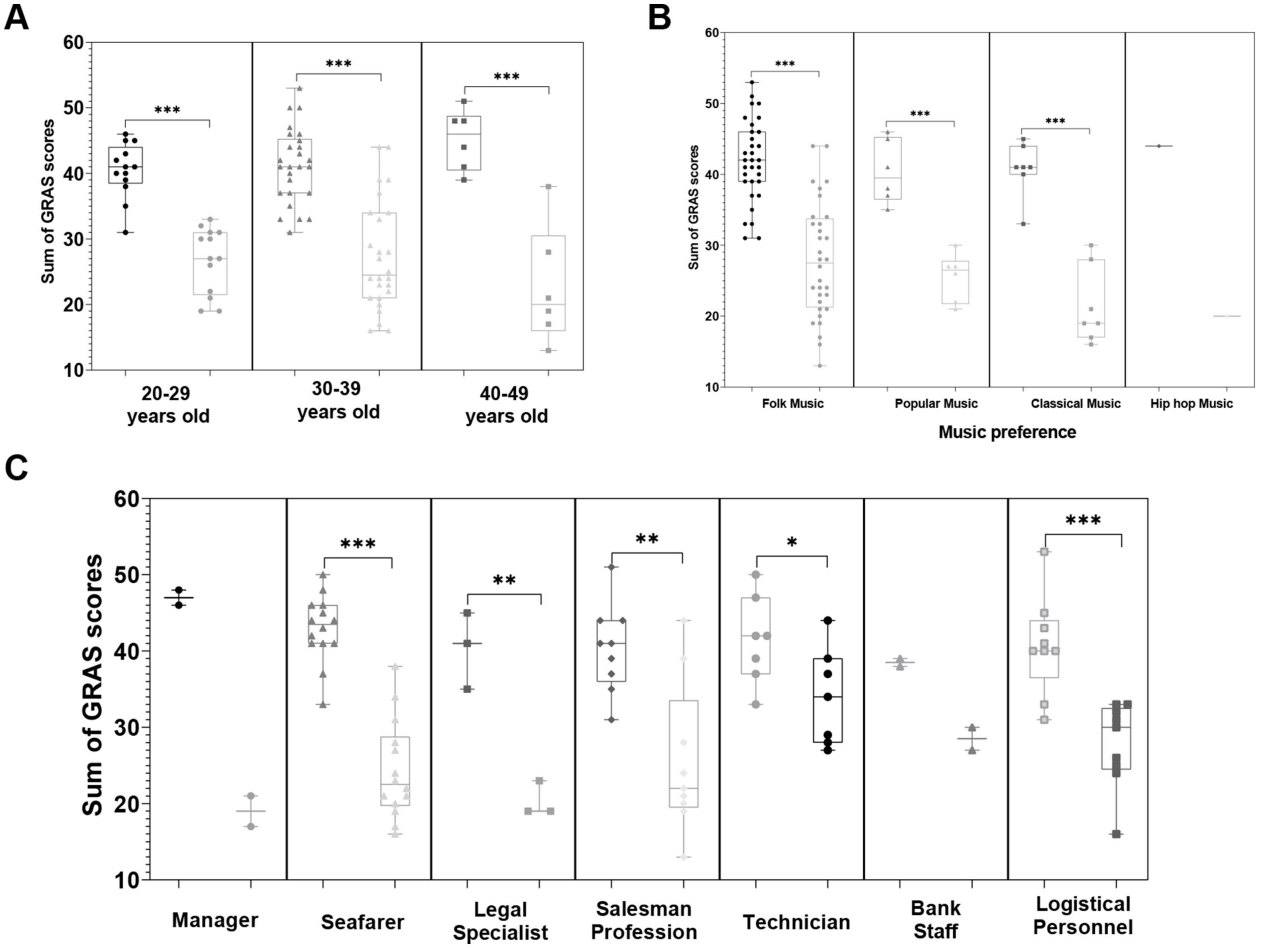
Impact of resource-oriented music therapy interventions on stress reduction across different age groups, music preferences, and occupational categories. **(A)** Violin plot illustrating stress reduction across age groups, with participants aged 40–49 showing the most significant improvement. **(B)** Grouped scatterplot highlighting stress relief effects based on music preferences, with participants preferring classical music demonstrating the most notable benefits. **(C)** The bar chart shows changes in stress across different occupational categories, with seafarers experiencing the most significant improvement in stress *p < 0.05, **p < 0.01, and ***p < 0.001.

Participants aged 40–49 showed the largest reduction in stress levels. The mean GARS score for this group decreased from 45.17 (SD = 4.62) before the intervention to 22.67 (SD = 9.00) after the intervention, with the median score decreasing from 44 to 20. The 20–29 age group showed a reduction from 40.46 (SD = 4.18) to 26.77 (SD = 5.00), and the 30–39 age group showed a decrease from 41.19 (SD = 5.70) to 27.35 (SD = 8.29). Figure 4B presents the distribution of stress scores across age groups. The 40–49 group demonstrated a visible narrowing of score variability, with most post-intervention scores concentrated in the lower range.

### Among different occupational groups, sailors show the most significant stress relief effect

Stress score changes were analyzed across different professions within the experimental group to examine the potential influence of occupational categories on the effectiveness of resource-oriented music therapy interventions (Table 5).

**Table 5:** Profession on therapeutic music listening in stress relief of overseas dispatched employees.

Occupation	Measurement stage	Number	Min	Max	25% Percentile	75% Percentile	Mean ± SD	S.E. Mean	LCL	UCL	Med
Manage	Pre-test	2	46	48	46	48	47 ± 1.14	1	46	48	47
	Post-test		17	21	17	21	19 ± 2.83	2	17	21	19
Seafarer	Pre-test	14	33	50	41	46	42.93 ± 4.36	1.165	41	46	43.5
	Post-test		16	38	19.75	28.75	24.36 ± 6.48	1.731	19	31	22.5
Legal	Pre-test	3	35	45	35	45	40.33 ± 5.03	2.906	35	45	41
	Post-test		19	23	19	23	20.33 ± 2.31	1.333	19	23	19
Sales	Pre-test	9	31	51	36	44	40.33 ± 5.81	1.936	35	44	41
	Post-test		13	44	19.5	33.5	25.56 ± 9.96	3.321	19	39	22
Technician	Pre-test	7	33	50	37	47	41.43 ± 5.80	2.192	33	50	42
	Post-test		27	44	28	39	34 ± 6.38	2.41	27	44	34
Bank employee	Pre-test	2	38	39	38	39	38.5 ± 0.71	0.5	38	39	38.5
	Post-test		27	30	27	30	28.5 ± 2.12	1.5	27	30	28.5
Logistics	Pre-test	9	31	53	36.5	44	40.67 ± 6.42	2.141	33	45	40
	Post-test		16	33	24.5	32.5	27.78 ± 5.61	1.869	24	33	30

LCL, Lower Confidence Limit; UCL, Upper Confidence Limit.

Seafarers showed the greatest reduction in stress levels, with mean GARS scores decreasing from 42.93 (SD = 4.36) before the intervention to 24.36 (SD = 6.48) after the intervention. The median score declined from 43.5 to 22.5. Managers showed a reduction from 47 (SD = 1.14) to 19 (SD = 2.83), sales staff from 40.33 (SD = 5.81) to 25.56 (SD = 9.96), and technicians from 41.43 (SD = 5.80) to 34 (SD = 6.38). Bank employees had the smallest change, decreasing scores from 38.5 (SD = 0.71) to 28.5 (SD = 2.12). Figure 4A visually represents the changes in stress scores across different occupational categories. Among these, seafaring work is characterized by extended time away from home, irregular schedules, and high-risk conditions, which are associated with a greater reduction in stress scores following the intervention.

### Among different music preferences, classical music enthusiasts experience significant stress relief

To investigate the impact of music preference on the effectiveness of resource-oriented music therapy interventions, this study analyzed the changes in stress scores before and after the intervention among participants with different musical preferences (Table 6). Participants who preferred classical music demonstrated the most significant stress reduction. Their pre-intervention mean stress score was 40.71 (SD = 3.86) with a median of 41, which significantly decreased to 21.43 (SD = 5.44) post-intervention, with a median of 19. This change was the largest among all music preference categories. In comparison, participants who preferred folk music showed a reduction from 41.91 (SD = 5.73) to 27.88 (SD = 8.13), while those who preferred pop music had their scores drop from 40.33 (SD = 4.46) to 25.5 (SD = 3.39). Only one participant preferred hip-hop music, and their stress score remained unchanged at 20 points before and after the intervention, making it difficult to draw any significant conclusions. Figure 4C further illustrates the influence of music preference on intervention outcomes, highlighting that participants who preferred classical music showed a notably greater improvement in stress relief when exposed to music that matched their personal preferences.

**Table 6:** Music preference on therapeutic music listening in stress relief of overseas dispatched employees.

Music preference	Measurement stage	Number	Min	Max	25% Percentile	75% Percentile	Mean ± SD	S.E. Mean	LCL	UCL	Med
Folk music	Pre-test	32	31	53	39	46	41.91 ± 5.73	1.013	39	45	42
	Post-test		13	44	21.25	33.75	27.88 ± 8.13	1.437	23	33	27.5
Pop music	Pre-test	6	35	46	36.5	45.25	40.33 ± 4.46	1.82	35	46	39.5
	Post-test		21	30	21.75	27.75	25.5 ± 3.39	1.384	21	30	26.5
Classical music	Pre-test	7	33	45	40	44	40.71 ± 3.86	1.459	33	45	41
	Post-test		16	30	17	28	21.43 ± 5.44	2.057	16	30	19
Hip hop music	Pre-test	1	44	44	44	44	44	—	—	—	44
	Post-test		20	20	20	20	20	—	—	—	20

LCL, Lower Confidence Limit; UCL, Upper Confidence Limit.

### Long-term effects of music-based and aerobic exercise interventions

To assess the long-term effects of the combined music-based and aerobic exercise intervention, follow-up evaluations of stress levels were conducted at 1 month, 3 months, and 6 months post-intervention (Table 7). The experimental and control groups achieved 100% retention throughout the six-month follow-up period (*n* = 46 per group).

**Table 7:** Changes in stress levels during follow-up (GARS scores).

Time point	Experimental group mean stress score (GARS)	Control group mean stress score (GARS)	Significance (*p*-value)	Change in experimental group (%)	Change in control group (%)
Baseline (pre-intervention)	3.60 ± 0.70	3.65 ± 0.65	–	–	–
Post-intervention (0 Months)	2.70 ± 0.60	3.50 ± 0.60	<0.001	↓25%	↓4%
Follow-up (1 Month)	2.75 ± 0.65	3.55 ± 0.62	<0.001	↓24%	↓3%
Follow-up (3 Months)	2.90 ± 0.70	3.60 ± 0.65	<0.01	↓19%	↓1%
Follow-up (6 Months)	3.05 ± 0.75	3.62 ± 0.68	<0.05	↓15%	↓1%

At the 1-month follow-up, the experimental group had a mean GARS score of 2.75 (SD = 0.65), slightly increasing compared to the post-intervention score of 2.70 (SD = 0.60), corresponding to a 1.85% change. The control group reported a score of 3.55 (SD = 0.62), similar to its baseline level of 3.65 (SD = 0.65). The between-group difference remained statistically significant (*p* < 0.001). At 3 months, the experimental group’s mean stress score increased to 2.90 (SD = 0.70), representing a 19.44% reduction from baseline. The control group scored 3.60 (SD = 0.65), nearly identical to its baseline. The between-group difference remained significant (*p* < 0.01). At 6 months, the experimental group had a mean stress score of 3.05 (SD = 0.75), a 15.27% reduction from baseline. The control group’s score was 3.62 (SD = 0.68), consistent with the initial measurement. Independent-sample t-tests indicated a statistically significant difference between groups at this time point (*p* < 0.05). All follow-up data were analyzed using complete cases without imputation for missing values.

## Discussion

Using a randomized trial design, this study systematically evaluated the effects of a combined music-based intervention and moderate-intensity aerobic exercise on stress levels among overseas employees. The results demonstrated that this intervention significantly reduced stress levels, with varying degrees of effectiveness observed across individual factors such as age, occupational category, and music preference. Participants synchronized their pace with musical rhythm during the exercise phase using metronome-guided audio. In the music listening phase, the volume and tempo of personalized music tracks were dynamically adjusted based on HRV, with calming tracks automatically selected when HRV decreased to enhance parasympathetic activation. The experimental group showed significantly lower GARS scores compared to the baseline at the end of the intervention, while the control group exhibited no similar improvements, indicating the substantial benefits of combining music and exercise interventions. Furthermore, a six-month follow-up revealed that the intervention effects were sustained over time, providing new empirical evidence for the long-term value of stress management strategies. These findings confirm the potential of music and exercise in the mental health domain and offer tailored psychological intervention strategies for overseas employees as a unique population. This research provides innovative stress management approaches for enterprises and policymakers, significantly improving overseas employees’ work efficiency and wellbeing.

The findings of this study align closely with the known physiological mechanisms underlying music-based and aerobic exercise interventions. Hillman et al. reviewed how aerobic exercise can enhance emotional regulation and cognitive function by promoting prefrontal cortex activation, neurogenesis, and the expression of brain-derived neurotrophic factor (37). The significant reduction in stress scores observed in the experimental group may reflect both psychological and physiological mechanisms. Although salivary cortisol samples were collected in this study, temperature-related degradation limited the analyzable dataset. Nevertheless, the observed stress reduction aligns with previous findings suggesting that music and exercise may modulate HPA axis activity and cortisol levels (19), while aerobic exercise contributes to emotional regulation by promoting the release of endorphins and dopamine (38). The persistence of the intervention effect, as evidenced by sustained reductions in stress scores at the 6-month follow-up, may reflect exercise-induced neuroplasticity, such as enhanced prefrontal cortex function (28).

In addition, the moderating role of personalized music preference may be explained through neurophysiological mechanisms. Participants who preferred classical music experienced greater stress relief, possibly because of its stable tempo and gradual harmonic progression are more likely to activate the default mode network and support emotional integration (17). These findings can be understood through a dual physiological-psychological perspective, where music and exercise jointly exert effects through cortisol reduction and neurotransmitter release, while personalized resource alignment, such as music preference, further enhances intervention outcomes via limbic system activation (39). This provides a theoretical foundation for the individualized design of stress management interventions.

Neuroscientific research suggests that music preferences like classical music may enhance emotional regulation by activating the mesolimbic reward system, including regions such as the nucleus accumbens and ventral tegmental area (39). EEG-based evidence further indicates that music interventions can improve prefrontal inhibitory control, which may account for the more pronounced stress reduction observed among participants aged 40–49 in this study, a group characterized by greater prefrontal cortex maturity (40, 41). Additionally, research on inter-brain synchrony in improvisational music therapy suggests that individuals in high-stress occupations, such as seafarers, may exhibit greater sensitivity to music-induced neural coupling due to prolonged social isolation, potentially contributing to stronger stress relief effects (42, 43). Additionally, Koelsch et al. systematically described how music activates the prefrontal cortex, limbic structures, and reward-related regions, highlighting its potential role in emotional regulation and the facilitation of social connectedness (44). These converging findings support the hypothesis that combined music and exercise interventions may induce stress reduction through multisystem neural processes, including emotional regulation, cognitive control, and social synchrony.

While the intervention effects observed in this study are consistent with previous findings, the design and outcomes offer several novel contributions. First, to our knowledge, this is the first randomized trial to evaluate the combined effects of music-based and moderate-intensity aerobic exercise interventions specifically targeting stress reduction in overseas employees—a population facing unique stressors related to cultural adaptation, occupational demands, and social isolation. Second, the study systematically examined the moderating roles of personalized factors such as age, occupational category, and music preference, offering theoretical support for tailored intervention design. Third, the six-month follow-up extended the time frame of evaluation beyond the short-term focus of most existing studies, providing insights into the sustainability of the intervention.

Despite its strengths, the study has several limitations. The overall sample size was relatively small, especially within specific occupational and music preference subgroups, which may limit the generalizability of the findings. Additionally, all interventions and assessments were conducted online, making it difficult to fully control external environmental influences. Finally, while the six-month follow-up adds value, longer-term outcomes remain unexplored.

Future research should consider larger and more diverse samples across cultural contexts to enhance generalizability. Longer intervention and follow-up periods are recommended to examine sustained effects and optimal intervention frequency. Neurophysiological methods such as resting-state fMRI and EEG hyperscanning could be used to explore the underlying mechanisms of personalized responses, particularly in relation to age and music preference. Integrating music and exercise interventions with other approaches, such as psychological counseling or cognitive behavioral therapy, may also help evaluate the synergistic effects of multimodal interventions.

The findings of this study offer practical implications for organizational management. Combining music-based and aerobic interventions can provide a cost-effective and accessible method for managing stress among overseas employees. Personalized intervention designs may further enhance acceptance and satisfaction, particularly in resource-constrained international work environments. This approach demonstrates strong potential for scalable implementation in similar occupational contexts.

In conclusion, this study provides the empirical evidence for the effectiveness of a combined music-based and aerobic intervention in managing stress among overseas employees. It highlights the moderating role of personalized factors and offers a novel, long-term perspective for non-pharmacological mental health interventions. These findings lay a scientific foundation for the development of more effective, culturally adaptive workplace stress management strategies.

## Conclusion

**Figure 5:**
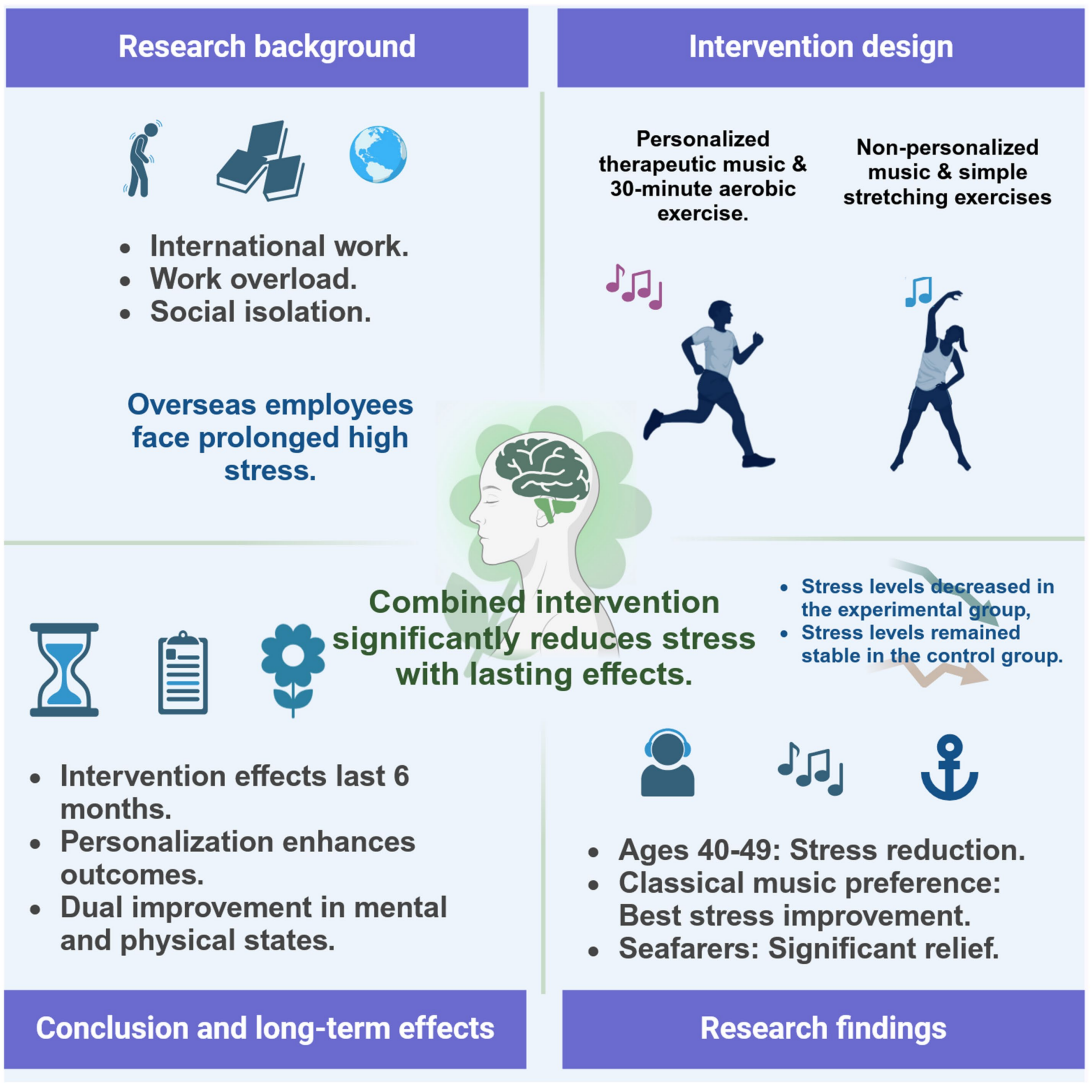
Mechanisms and effects of the combined resource-oriented music therapy and aerobic exercise intervention in significantly reducing stress among overseas employees.

In summary, the following preliminary conclusions can be drawn: combining music-based interventions and moderate-intensity aerobic exercise significantly reduces stress levels among overseas employees (Figure 5). The intervention is particularly effective for participants aged 40–49, those who prefer classical music, and individuals in high-pressure occupations (e.g., seafarers). The combined intervention demonstrates immediate stress relief effects and sustained long-term benefits. This study supports the feasibility and provides preliminary evidence for effectiveness of personalized and multidimensional interventions in psychological management for high-stress occupational groups.

## Data Availability

The original contributions presented in the study are included in the article/Supplementary material, further inquiries can be directed to the corresponding author.

## Supplementary material

SUPPLEMENTARY FIGURE S1 Adjusted GARS.
