# Supplementary figures and images for: Integrative stress management for global workforce: music-based and exercise intervention for overseas employees
Source: Front Public Health. 2025 Nov 6;13:1603059. doi: 10.3389/fpubh.2025.1603059 (PMC12633643; doi:10.3389/fpubh.2025.1603059)

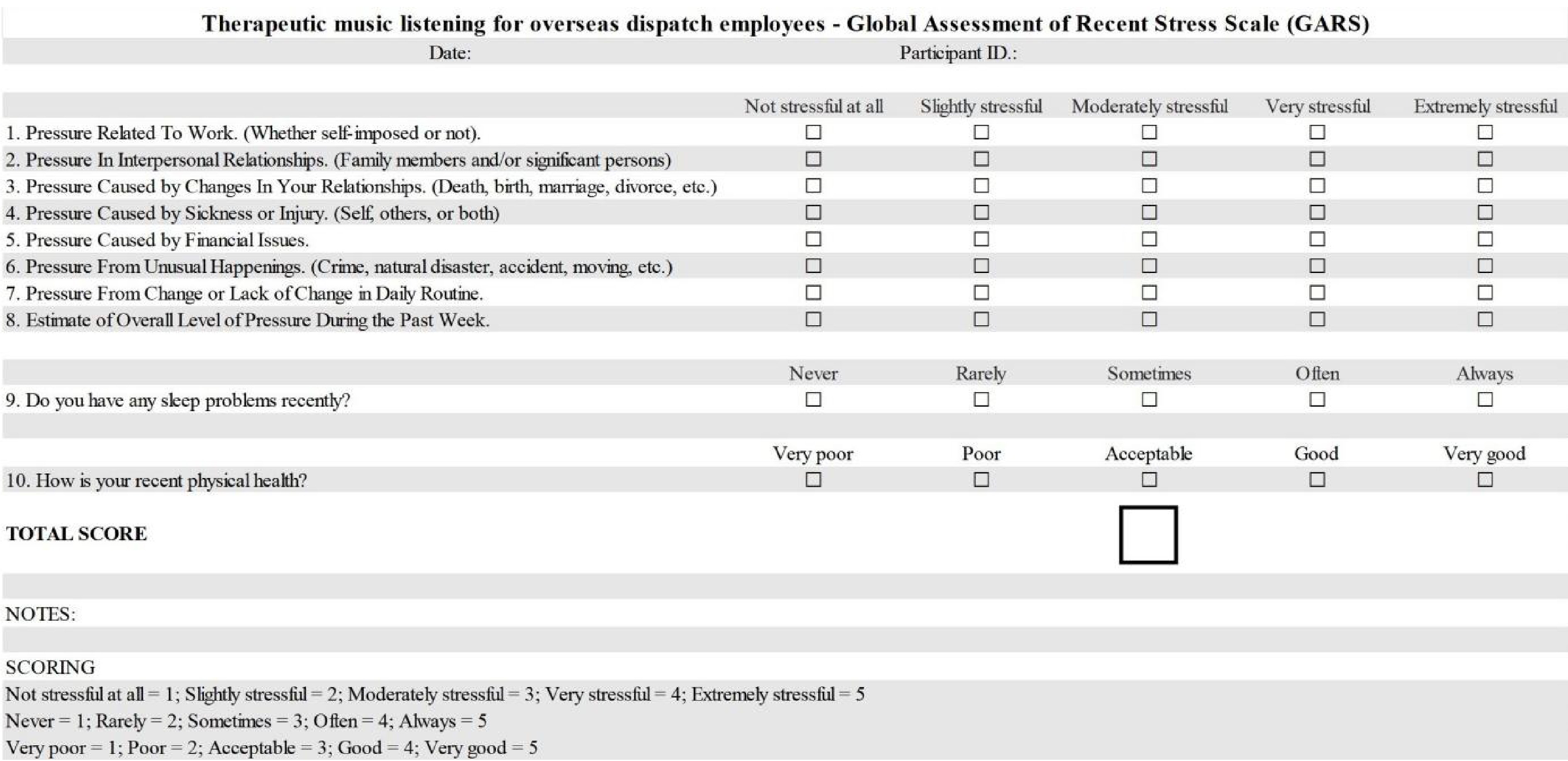

Supplement: SUPPLEMENTARY FIGURE S1 — Adjusted GARS. [file Image_1.JPEG]
