# Supplementary material for: Integrative stress management for global workforce: music-based and exercise intervention for overseas employees
Source: Front Public Health. 2025 Nov 6;13:1603059. doi: 10.3389/fpubh.2025.1603059 (PMC12633643; doi:10.3389/fpubh.2025.1603059)
Supplement: Supplementary file 2 [file Table_1.docx]

# Table S1 Sample of Background Music Used in Control Group

|  | **Song** | **Composer / Source** | **Musical Instrument** | **Duration** | **Source** | **Speed** | **Beat** |
| --- | --- | --- | --- | --- | --- | --- | --- |
| 1 | Gymnopédies No.1 | Erik Satie | Piano | 3:25 | Public Domain | 72 | 3/4 |
| 2 | Rainforest Ambience | Nature Sounds Archive | Ambient (Rain, Birds) | 5:00 | Field Recording | N/A | N/A |
| 3 | River Stream Flow | BBC Nature FX | Ambient (Water Flow) | 4:30 | BBC | N/A | N/A |
| 4 | Meditation Piano 1 | Kevin MacLeod | Piano | 3:45 | Incompetech | 76 | 4/4 |
| 5 | Ocean Waves | FreeSound | Ambient (Waves) | 4:10 | FreeSound.org | N/A | N/A |
| 6 | Claire de Lune | Claude Debussy | Piano | 4:50 | Public Domain | ~66 | 3/4 |
| 7 | Morning Mood | Edvard Grieg | Orchestra | 3:45 | Public Domain | ~78 | 6/8 |
| 8 | Calm Forest Wind | Binaural Nature | Ambient (Wind, Leaves) | 5:15 | Nature Archive | N/A | N/A |
| 9 | Light Ambient Piano | Bensound | Piano | 4:05 | Bensound.com | 75 | 4/4 |
| 10 | Soft River and Birds | Relaxing Nature | Ambient (Birds + Stream) | 5:30 | Relaxing Sounds | N/A | N/A |

# Table S2. Sample of Background Music Used in Experimental Group

|  | **Song** | **Composer / Source** | **Musical Instrument** | **Duration** | **Source** | **Speed** | **Beat** |
| --- | --- | --- | --- | --- | --- | --- | --- |
| 1 | 1974, Way  Hom | Mondo  Grosso | Piano，Drum | 4:20 | MG4 | 80~85 | 4/4 |
| 2 | True | Martin | Guitar | 3:52 | solo | 75~85 | 4/4 |
| 3 | Hong yan | Orchestra | SymphonyOrchestra  cello | 4;21 | solo |  | 4/4 |
| 4 | Pipa language | Zide Qin Society | Guqin  gangshe  drum | 3:18 | ensemble | 60~120 | 2/4 |
| 5 | Shuimolanting | lizhihui | Guzheng | 5:34 | solo | 60~70 | 4/4 |
| 6 | Uta Mischa Maisky Silent Woods | Tamezo  Narita  Hamabe no | Cell,piano | 3:32 | ensemble | 84~86 | 3/4 |
| 7 | One flower one world | lizhihui | Zhudi | 5;50 | solo | 60~70 | 4/4 |
| 8 | Anheqiao | Song dongye | Guitar, matouqin | 2;40 | ensemble | 80~85 | 2/4 |
| 9 | Pugongying de yueding | Joy, zhou | piano, cello | 3;40 | ensemble | 75~85 | 2/4 |
| 10 | Funf Stucke Im | Robert  Schumann | orchetra | 4:44 | Solo | 74 | 2/4 |

**Table S3. Resource~Oriented Music Therapy Method and Process.**

| **Stage** | **Treatment goals** | **Work content and use of technology** |
| --- | --- | --- |
| Preparatory stage | Preliminary investigations on employee data | optional registration of participationcollect personal informationSign an agreement to participate in the experiment |
| Preliminary assessment | Initial stress assessment of treated population | Global Assessment of Recent Stress (GARS) |
| Music intervene | Intervention process #1 #2 #3 #4 #5 | Find inner resourcesShare musicChoose to match the inner resources musicResource orientation guidance |
| Reassessment | Reassess the client's stress level | Global Assessment of Recent Stress (GARS) |
| Efficacy assessment | Participation process and resource orientation survey | One~on~one interview |
